# Editorial: Multiomics approaches for understanding autism spectrum disorder

**DOI:** 10.3389/fnins.2025.1542260

**Published:** 2025-02-11

**Authors:** Jernej Kovac, Branko Aleksic, Danijela Krgovic

**Affiliations:** ^1^Clinical Institute of Special Laboratory Diagnostics, University Children's Hospital, University Medical Centre, Ljubljana, Slovenia; ^2^Graduate School of Medicine, Nagoya University, Nagoya, Aichi, Japan; ^3^Clinical Institute of Genetic Diagnostics, University Medical Centre Maribor, Maribor, Slovenia; ^4^Department of Molecular Biology, Faculty of Medicine, University of Maribor, Maribor, Slovenia

**Keywords:** autism spectrum disorder (ASD), multiomics, neuroepitranscriptomics, RNA sequencing (RNAseq), artificial intelligence (AI)

Autism Spectrum Disorder (ASD) is an intricate neurodevelopmental condition encompassing an array of genetic, molecular and behavioral traits. The Research Topic, “*Multiomics Approaches for Understanding Autism Spectrum Disorder*” marries investigations that delve into the intricacies of ASD with the goal of enhancing our comprehension of its roots and impacts while proposing tailored treatment strategies.

Wu et al.'s research on SHANK2 variants presents a progress, in comprehending the causes of ASD. The discovery of novel SHANK2 variants enriches the range of identified harmful mutations and emphasizes the significance of impaired synaptic functions in the development of ASD. By incorporating RNA sequencing this study provides an insight into irregular gene expressions focusing on pathways related to synapses and protein binding, in ASD. This research emphasizes the potential therapeutic approach of targeting mechanisms for synaptic stabilization.

Lee et al. explore the growing field of neuroepitranscriptomics, highlighting how RNA modifications like m6A and m3C influence the growth and adaptability of neurons. Their research sheds light on how disruptions in RNA modifications can affect functions and synaptic communication linking these changes to developmental disorders such as ASD. This study not only enriches our knowledge about the roles of RNA but also proposes new molecular targets for potential interventions.

Deng et al. investigate how disrupted neural connections impact individuals, with ASD, examining areas such as the medial prefrontal cortex, and amygdala, key for social cognition. By utilizing Granger Causality Analysis they uncover reduced connectivity in children with ASD linking these shortcomings to difficulties in social preference. These results open avenues for using imaging biomarkers in diagnosing and tracking ASD over time.

Mongad et al.'s advanced literature mining system demonstrates how AI can expedite research by categorizing insights from extensive data sets. Their multiomics study emphasizes the fusion of genomics, transcriptomics and proteomics, in defining braineight molecular landscape underscoring the importance of interdisciplinary approaches to unraveling its complex characteristics.

Collectively, these studies highlight the potential, of multiomics approaches in autism research. The range of genetic variants, including SHANK2 underscores the importance of large scale studies to discover both rare and common mutations associated with autism. The emerging discipline of neuroepitranscriptomics showcases the promise, of targeting RNA pathways to address neurodevelopmental issues. Additionally linking neural network changes with behavioral traits provides a strong foundation for creating interventions guided by neuroimaging. Lastly tools like literature mining pipelines demonstrate how computational techniques can gather knowledge, from various fields, to accelerate research in applications. This Research topic showcases how multiomics can help understand the aspects of ASD emphasizing the disorders complexity and the potential of tailored medicine. Researchers are getting closer to uncovering ASDs secrets by combining genomic, epigenomic and functional information with the goal of enhancing diagnosis and treatment results for those affected. These discoveries not drive progress in the field. They also underscore the need for teamwork, bringing together various fields of science to tackle one of the challenging neurodevelopmental issues of our time.

